# Tipping points and early warning signals in the genomic composition of populations induced by environmental changes

**DOI:** 10.1038/srep09664

**Published:** 2015-05-12

**Authors:** Jacobo Aguirre, Susanna Manrubia

**Affiliations:** 1 Centro de Astrobiología (INTA-CSIC), ctra. de Ajalvir km 4, 28850 Torrejón de Ardoz, Madrid, Spain; 2 Centro Nacional de Biotecnología, CSIC, c/Darwin 3, 28049 Madrid, Spain; 3 Grupo Interdisciplinar de Sistemas Complejos (GISC), Madrid, Spain

## Abstract

We live in an ever changing biosphere that faces continuous and often stressing environmental challenges. From this perspective, much effort is currently devoted to understanding how natural populations succeed or fail in adapting to evolving conditions. In a different context, many complex dynamical systems experience critical transitions where their dynamical behaviour or internal structure changes suddenly. Here we connect both approaches and show that in rough and correlated fitness landscapes, population dynamics shows flickering under small stochastic environmental changes, alerting of the existence of tipping points. Our analytical and numerical results demonstrate that transitions at the genomic level preceded by early-warning signals are a generic phenomenon in constant and slowly driven landscapes affected by even slight stochasticity. As these genomic shifts are approached, the time to reach mutation-selection equilibrium dramatically increases, leading to the appearance of hysteresis in the composition of the population. Eventually, environmental changes significantly faster than the typical adaptation time may result in population extinction. Our work points out several indicators that are at reach with current technologies to anticipate these sudden and largely unavoidable transitions.

The identification of indicators signalling the proximity of a critical transition in the dynamics of natural systems is an emerging field of research^1^. Early-warning signals have been described in widely different systems which, however, share important architectural features. These include a global network of connections between components and a non-linear response to change promoted by positive feedbacks^2^. Often, those studies rely on simple models that have educated our intuition and clearly delineated the framework where tipping points occur, many of them focusing on climate dynamics^3^,^4^. The conceptual tools at hand have been applied to characterize *a posteriori* sudden shifts of state in ecosystems^5^, physiology, and finance^2^, and are beginning to be used to identify tipping points in *in vitro* natural populations^6^. In all those examples, the system dynamics proceeds under the effect of external changes that implicitly modify the fitness landscape. Indeed, the fitness value of a given population is context dependent, and thus varies concomitantly with the environment: eventually, sudden changes of state occur. Among several other well-documented examples in ecology we find the loss of coverage by charophyte vegetation experienced by lake Veluwe in response to the increase in phosphorus concentration^7^. The sudden decrease observed at a critical concentration in the early 1970s was later reverted in the 1990s as the concentration of phosphorus decreased, though that occurred through a hysteresis cycle. In single populations of unicellular organisms^6^, an instability separating a parameter region leading to a stable fixed point of high population density from a region ushering in extinction appears as a consequence of variations in population density. That experiment can be understood as a case of a slowly driven landscape that causes systematic decreases in the population density. In a fitness landscape representation of this second example, the lower the population density the lower its fitness. The population is pushed towards a valley and recovers with increasing difficulty until, at a critically low density, the attractor of the dynamics shifts to the extinct state.

Recent studies posit that smooth alterations of the environment might be responsible for some of the drastic state shifts that have been reported in our planet's history, and warn about the repetition of this phenomenon in the nearby future^2^,^8^,^9^. Sudden shifts in the states of evolving populations interrupting long periods of stagnancy have been described at the level of species^10^, bacteria^11^ and viral genomes^12^. Several models have interpreted those transitions as jumps along evolutionary optimization, with stasis periods due to entropic or fitness barriers that the population has difficulty in overcoming^13^,^14^,^15^, or as selective sweeps leading to a new phenotype^12^. Here we analyse these transitions in realistic, slowly changing fitness landscapes of genomes, and show that it is a generic phenomenon. Though most populations experience sudden genomic shifts in out-of-equilibrium situations, as those described above, this behavior also occurs when populations are allowed to attain mutation-selection equilibrium between perturbations (either stochastic or driven), as we here show. Several indicators quantify the nearness of the transition.

In Methods we present a general model to study population dynamics on stochastic landscapes. In particular, we devise a representative example of a broad class of models for evolving populations in fixed environments. The fitness landscape is generated through the NK model of Kauffman^16^, which yields landscapes of tunable ruggedness with properties characteristic of natural systems: multiple peaks, epistatic interactions, and local optima^17^. The landscape is a network of genomes mutually accessible through mutations. It is endowed with a fraction of lethal or strongly deleterious mutations, in agreement with observations^18^. Finally, we pay special attention to the mathematical description of the population dynamics over the fitness landscape, and the relevant quantities that characterize it.

Evidence in favour of a direct effect of environmental conditions in fitness landscapes is mounting^19^,^20^,^21^. In Results we analyse time-varying fitness landscapes by implementing environmental perturbations through the addition of stochastic noise to the fitness value of genomes. We focus on two types of varying environments: (i) a constant fitness landscape affected by stochastic fluctuations; and (ii) a driven fitness landscape. The latter is motivated by the fact that driving forces in the history of our planet leading to global changes, such as temperature, concentration of oxygen, carbon dioxide or hydrogen sulphide, changed smoothly but monotonically — in many cases almost linearly — with time before tipping points were reached^2^,^8^. We prove analytically and characterize numerically that sharp transitions at the genomic level preceded by early-warning signals are a generic phenomenon in constant and slowly driven landscapes affected by even slight stochasticity, and focus on the robustness of the phenomenon described. In the Discussion we analyse the generality of the results obtained, their influence in our understanding of complex ecological systems, and present a protocol based on current high-throughput sequencing technologies to anticipate these sudden genomic transitions whose consequences are not fully understood yet.

## Methods: General model for population dynamics on stochastic landscapes

### The stochastic fitness landscape

A genome is defined as a sequence of length *N*. The state of each locus is taken from an alphabet of *A* letters. The space of genomes is a regular network of size *m* = *A^N^* and dimension *N* where two nodes (or genomes) are connected by an undirected link if they differ in the state of only one locus. Each node has (*A* − 1)*N* neighbours or connections to other genomes. This quantity defines its degree. The maximum distance between two sequences is *N*. When *A* = 2 the space of genomes is a hypercube of dimension *N*.

The *NK* model is based on the spin glass model of statistical physics^22^, and represents one of the most versatile models to map genotypes to rugged fitness landscapes^16^,^23^,^24^. It represents genomes with *N* loci (genes, nucleotides, aminoacids, etc.), that take one out of *A* different states (number of alleles, nucleotides, aminoacids, etc.) Epistasis, or interaction between loci, is defined here as an interaction of each locus with *K* other loci of the same genome. The larger *K*, the more rugged the landscape (see below).

The fitness value *f_s_* of a genome *s* in a given fitness landscape 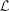 is calculated as follows (for the sake of clarity we have presented a detailed example in section S1 of the Supplementary Information). First, we construct a matrix **L** of size *A^K^*^ + 1^ × *N* whose elements *L_ij_* are random numbers uniformly chosen between 0 and 1. Elements in column *j*, 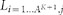, represent all possible contributions of locus *j* to the total fitness of the genome *s*. The actual contribution of locus *j*, 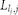, depends on its state and on those of the *K* other loci with which it interacts. For this reason, there are *A^K^*^ + 1^ values in each column, representing the *A^K^*^ + 1^ possible combinations of *K* + 1 elements taking *A* different states (and index *l_j_* stands for the correct one for locus *j*).

The fitness of sequence *s* is the average value of the fitness contributions of each of its loci,
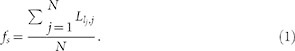
Note that, due to the normalisation above, all fitness values satisfy 0 ≤ *f_s_* ≤ 1. As matrix **L** is stochastically generated, the corresponding fitness landscape 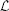 is different every time we run the algorithm. Varying parameter *K* yields fitness landscapes with features akin to the properties measured in different natural genome spaces (DNA, RNA, proteins, etc.)^25^. Low values of *K* give rise to smooth Fujiyama landscapes, that is, landscapes with only one high peak and where similar genotypes show similar fitness values. High values of *K* describe rugged landscapes with many local maxima and where close genotypes may show very different fitness, the more different the higher *K*. In the upper limit *K* = *N* − 1, the fitness of each genotype is not correlated at all with that of any other genotype. Parameter *K* thus characterises landscape ruggedness.

Finally, the existence of sequences carrying lethal mutations, that is with fitness zero, cannot be discarded in realistic fitness landscapes: Natural landscapes are holey^26^ and a significantly large fraction of all possible mutations leads to non-viable individuals^18^. These two features result in actual genome networks that are topologically heterogeneous.

Lethal mutations are introduced in the model as follows. First, we generate the *NK*-fitness (*f*_0_)*_s_* of each sequence *s* as explained above. Then, we introduce the lethality coefficient, a new parameter 0 ≤ *f_l_* ≤ 1 such that those genotypes with (*f*_0_)*_s_* > *f_l_* are defined as lethal and assigned fitness zero (*f_s_* = 0), and the remaining values are rescaled: if (*f*_0_)*_s_* > *f_l_*, then *f_s_* = (*f*_0_)*_s_* − *f_l_*. A fraction of lethal genomes is so generated, whose number has to be calculated after the truncation of the landscape has been applied.

This procedure immediately yields a heterogeneous network, now formed by nodes with degree between 0 and the maximum value (*A* − 1)*N*. In the limit *K* = *N* − 1, the fitness of each sequence is independent of its neighbours and the lethal mutations so introduced would thus be randomly distributed. For any *K* > *N* − 1 correlations exist and our procedure yields connected groups of genomes larger than randomly expected and also clustering in the distribution of lethal mutants. Both facts are in agreement with the existence of mutational hot spots (regions in the genome that tolerate mutations) and highly conserved sequences (regions where mutations likely cause deleterious effects and are eliminated by selection).

### Mathematical description of population evolutionary dynamics in a fixed fitness landscape

The evolutionary dynamics of a population replicating and mutating in a networked, fixed fitness landscape, can be cast in mathematical form^27^,^28^. For the sake of completeness, here we present its main properties applied to the present situation.

A wide variety of evolutionary processes occurring in a network of *m* nodes can be mathematically described as
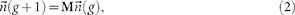
where 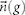 is a vector whose components are the population of individuals at each node at generation (or time) *g* and **M**, with *M_ij_* ≥ 0, is a transition matrix of dimension *m* × *m* that contains all details on the evolutionary process (note that *m* = *A^N^* in our model).

**M** is a primitive matrix. For this reason, its largest eigenvalue is positive, it verifies that *λ*_1_ > |*λ_i_*|, ∀ *i* > 1, and the elements of its associated eigenvector 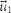 are also positive. Therefore, the dynamics can be written as
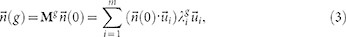
where 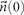 is the initial condition and 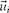 the *i*-th eigenvector of **M**, *i* = 1, …, *m*. Eq. (3) reveals that the system evolves towards an asymptotic state independent of the initial condition and proportional to the first eigenvector 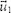. Its associated eigenvalue *λ*_1_ yields the growth rate at the asymptotic equilibrium. If 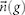 is normalised such that 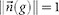 after each iteration (the population is kept constant), 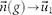 when *g* → ∞.

As explained above, strictly speaking the number of generations elapsed before equilibrium is reached is infinity. However, there is a way we can define an effective time to equilibrium *g_e_*. We define *g_e_* as the number of generations required for 
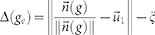
, where *ξ* ≪ 1 is a tolerance. In all cases where 0 ≤ *λ*_3_ > *λ*_2_ and 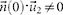, *g_e_* can be calculated from Eq. (3) and Δ(*g_e_*) through the excellent approximation
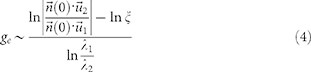
thanks to the exponentially fast suppression of the contributions of higher-order terms (*λ_i_* ≥ *λ_i_*_ + 1_, ∀*i*; see^27^).

Let us focus on the description of the current model. The explicit form of the transition matrix **M** depends on the particular system studied, but it is always a function of the mutual accessibility of genotypes and their fitness. These two quantities are represented through (i) the adjacency matrix **G**, which encodes the regular topology of the space of genomes; elements of **G** are *G_ij_* = 1 if nodes *i* and *j* are connected and *G_ij_* = 0 otherwise, and (ii) diagonal matrix **F** containing the fitness value of each node obtained following the former section, *F_ij_* = *f_i_δ_ij_*, where *i*, *j* = 1, …, *A^N^*.

For instance, a population whose individuals produce *r* offspring per generation that mutate with a probability *μ* per genome and replication cycle, and which substitute the parental population, evolves according to the transition matrix
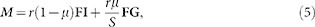
where *S* ≡ (*A* − 1)*N* is the total number of neighbours of each genome. Note that the effective reproduction rate of genome *i* is *rf_i_* due to the fitness factor. However, it is important to emphasise that the only quantity capturing the growth rate of the population at equilibrium (and thus the replication rate of all sequences in the population) is *λ*_1_, the largest eigenvalue of matrix **M**.

In the particular case studied here, we have chosen *μ* = 1 to highlight the role played by the second term, which contains all information on the interplay between fitness and topology. Also, mutation rates of the order of one change per genome and replication cycle are not rare^29^, so this situation is representative at least of fast mutating organisms.

In general, the growth of the population at equilibrium is given by the largest eigenvalue *λ*_1_. Hence, *λ*_1_ > 1 implies exponential growth, *λ*_1_ = 1 stands for a constant population size, and *λ*_1_ > 1 means a shrinking population. Note that the *NK* model, by definition, produces values of *F_ij_* > 1. Therefore, *r* should be large enough so as to avoid extinction. In general, indeed, fitness values are arbitrary as long as they are larger or equal than zero, so they admit a rescaling by any constant factor which, if large enough, would eventually yield *λ*_1_ > 1. As explained above, this rescaling (here represented through *r*) does neither modify the eigenvectors of matrix **M**, nor the dynamics of the population. In order to avoid maintaining a meaningless factor in front of our dynamical equations, and without loss of generality, we will study the case
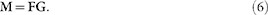


In the case of the *NK* model, as discussed, *λ*_1_ > 1, but we could simply rescale the height of the fitness landscape by a constant arbitrary factor (subsumed in *r*) without causing any other quantitative change. For completeness, in section S2 of the Supplementary Information we will show that all the phenomenology obtained for Eq. (6) is even quantitatively enhanced for values of the mutation rate *μ* > 1 and using the more general Eq. (5).

The transition matrix defined by Eq. (6) is symmetrizable and all its eigenvalues *λ_i_* are real^27^. However, **M** is not symmetric, because of its dependence with **F**. As a result, there is no simple relationship between the eigenvalues and eigenvectors of the symmetric matrix **G** and those of **M**: topology and fitness cannot be decoupled.

### Definition of relevant quantities in fixed fitness landscapes

In addition to the quantities already introduced, as the number of generations to equilibrium *g_e_*, and all those characterising the global dynamics as *λ_i_* and 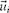, we introduce in the following a number of variables that quantify the evolutionary process and some important changes in its nature. Let us remark that though the quantities presented below have been defined at mutation-selection equilibrium, that is, for *g* → ∞, they can be generalised for any time *g* by substituting 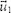 by the corresponding state of the population 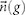.

#### Matrix of minimum distances between genomes

The fitness landscape we are investigating has a fraction of sequences with zero fitness. They cause holes in the landscape, which correspond to regions that cannot be traversed by the population. We now define matrix **D** as a matrix of distances between genomes. Its elements *D_ij_* yield the minimum path between sequences *i* and *j* when linked through the adjacency matrix but avoiding sequences with zero fitness. In other words, the value of *D_ij_* is the minimum number of mutations that permit to move from genome *i* to genome *j* by single mutations through a path of positive fitness.

#### Genetic distance at equilibrium

In order to quantify the position of the population in the fitness landscape at equilibrium, we have introduced the genetic distance *d*_0_. This distance evaluates how far, according to **D**, each of the genomes currently represented in the population is from an arbitrary reference point. As reference we have chosen sequence 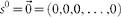. The total distance is the sum over all individuals. In mathematical form,
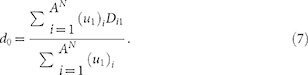
The first eigenvector of the transition matrix **M**, 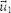, represents the population at equilibrium and assigns the corresponding weight to each sequence (subindex *i* stands for the *i*–th component of the vector); matrix **D** has been ordered in such a way that its first column contains the distances of all nodes in the landscape to sequence *s*^0^. The normalised product of those two quantities defines the genetic distance *d*_0_.

#### Average fitness of the population at equilibrium

The fitness value of node *i* in the landscape is *f_i_*. Consequently, the fitness associated to such landscape is 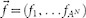, and the average fitness of the population at equilibrium, 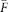, is
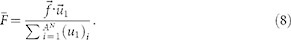


### Definition of relevant quantities in time-varying fitness landscapes

Up to here, we have focused on the dynamics of populations on fixed landscapes. However, in natural environments landscapes evolve in time. The following quantities measure the evolution of the population distribution over the space of genomes and that of the fitness landscape in a varying environment.

#### Difference to initial equilibrium

In a varying environment we might be interested in the difference between the state of the population at the equilibrium or the fitness landscape at a given environment *τ* and in the original situation *τ* = 1. The difference between the mutation-selection equilibrium of the population in the initial landscape and the equilibrium attained in a later environment *τ* is quantified as
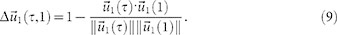


The difference between the initial fitness landscape and that at environment *τ* is similarly calculated,
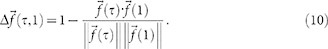


#### Difference between subsequent equilibria

Similarly to the former definitions, in a varying environment the difference of the state of the population at the equilibrium at two contiguous environments *τ* and *τ* + 1 is precisely defined as
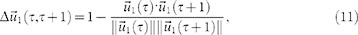
and the corresponding relative change in fitness landscape is
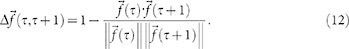


Note that the difference to initial condition (Eqs. (9) and (10)) and the difference between subsequent equilibria (Eqs. (11) and (12)) are defined in^0,1^, where zero and one stand respectively for a total similarity and an absolute dissimilarity between the composition of the population and the fitness landscape at the two compared environments.

## Results

Fitness values are strongly influenced by the environment. Fitness is a context-dependent quantity, and multiple causes modify the environmental conditions of ecosystems. Inspired by two common scenarios, we have implemented the following situations where fitness changes are effectively affected by the environment: (i) a stochastically varying fitness landscape and (ii) a driven fitness landscape.

### Stochastically varying fitness landscape

We first consider a randomly fluctuating landscape. For this purpose, we calculate a starting fitness landscape 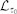, whose associated random matrix is 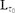, in the way shown in Methods. The elements of the random matrices **L***_τ_* associated to subsequent fitness landscapes 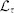 are obtained as follows
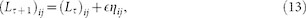
where *η_ij_* is a random number uniformly distributed in [−1, 1] and 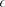 is the environmental noise intensity. We use the variable *τ* to order the fitness landscapes where the population will evolve. Note that it is not a proper time, but a label used for convenience to indicate the succession of environments.

The fitness of all sequences is obtained as described in Methods. Our procedure maintains the expected correlation, given *K*, between the fitness of neighbouring genotypes, since the stochastic variation is added to the elements of matrix **L***_τ_* and not to the values *f_s_*.

**Figure 1:**
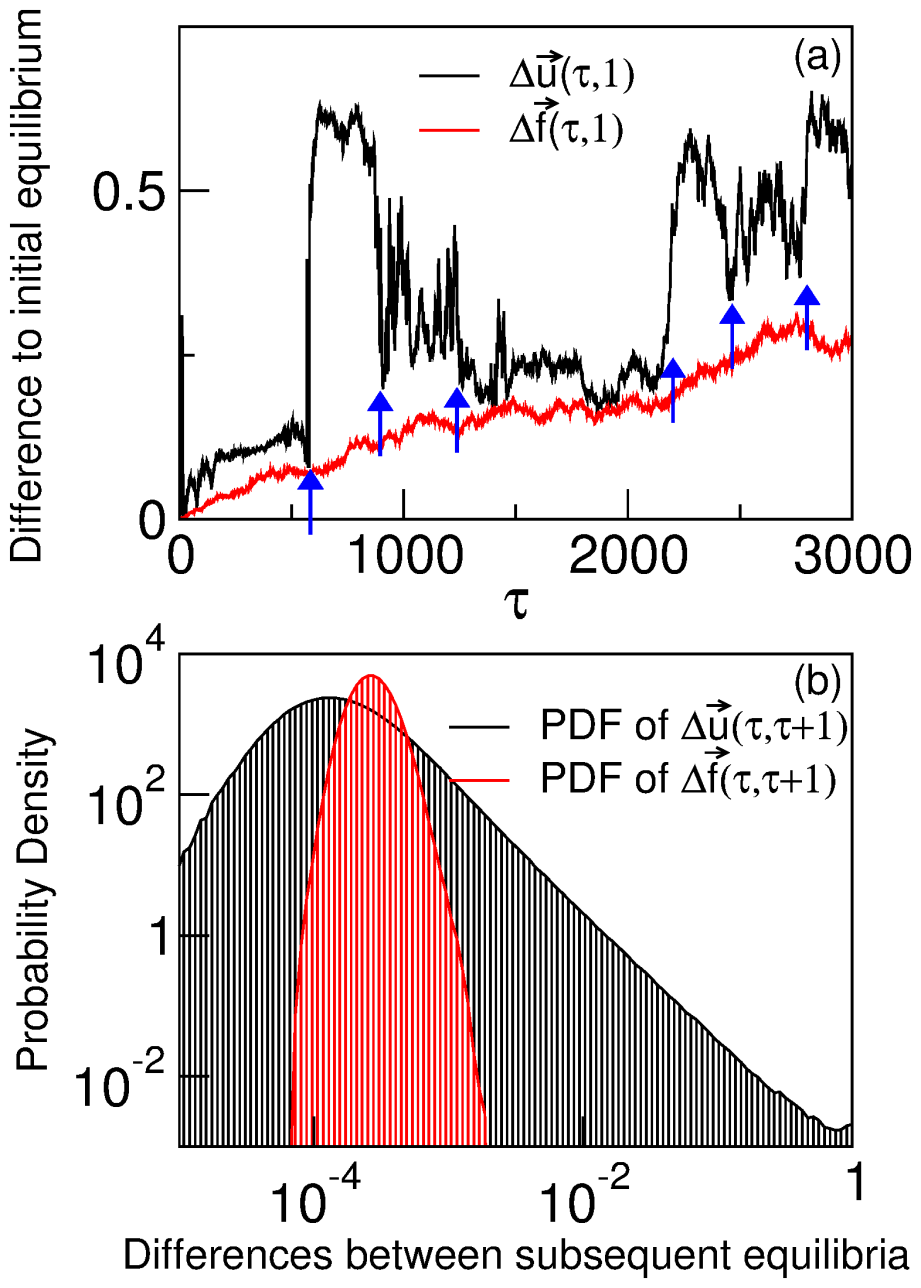
Mutation-selection equilibria in a stochastically varying environment. (a) Difference of the state of the population (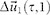, black) and the fitness landscape 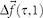, red) to the initial environment *τ*_0_ = 1. The fitness landscape drifts randomly as small stochastic perturbations accumulate, while the position of the population flickers. The *x*–axis represents contiguous landscapes which accumulate slight changes from *τ* to *τ* + 1. The arrows mark some of the sudden transitions in the population distribution. (b) Histograms of differences between the state of the population (black) and fitness landscapes (red) in subsequent equilibria *τ* and *τ* + 1. A series of 2 · 10^6^ consecutive fitness landscapes has been used. While the distribution of changes in the landscape is narrow, that of changes in the population state has a power-law-like tail. Parameters are *N* = 8, *K* = 4, *f_l_* = 0.55, with an alphabet of two letters and environmental noise 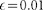.

In Fig. 1 we calculate the mutation-selection equilibrium (*g* → ∞) of a genomic population with sequences of length *N* = 8 and a two-letter alphabet, for a fitness landscape 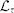 that evolves stochastically as explained above. It turns out that the population distribution in subsequent environments can be deeply dissimilar to that of the initial environment for fitness landscapes affected by little change. Figure 1(a) plots the difference of the population distribution at environment *τ* to the initial equilibrium, 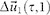 (Eq. (9)), and that of the fitness landscape 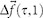 (Eq. (10)). The environmental noise of small amplitude causes flickering in the genomic composition of populations even when the time scales of adaptation and environmental change are decoupled. This strongly non-linear response of the population is captured by the histograms of 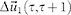 (Eq. (11)) and 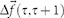 (Eq. (12)), the differences between the population and fitness state in the equilibria at contiguous environments *τ* and *τ* + 1 (Fig. 1(b)). While the distribution of changes in the landscape is narrow, that of changes in the population state shows a power-law-like distribution, highlighting the generality of the abrupt transitions marked by arrows in Fig. 1(a).

But how general is the presence of these genomic shifts when we vary the parameters, and which is the reason for their appearance? In section S3 of the Supplementary Information we calculate how the variations in the fitness and population of a node are distributed in the equilibrium at contiguous environments *τ* and *τ* + 1, following the model presented here. We provide a proof of the qualitative difference between both distributions: the stochastic variable characterising changes of fitness in a single node has a Gaussian, narrow distribution, while the response of the population of that node to changes in fitness can be arbitrarily large. Hence, the distribution corresponding to the latter variable can develop a long, fat tail. The calculations yield:
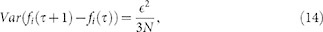

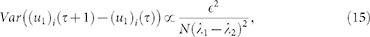


that is, the variance of changes in the fitness of each genome *i* of length *N* is proportional to the squared noise amplitude 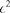 (and thus is small as far as the noise intensity is also small), while that of changes in its population can grow notably because it is inversely proportional to the spectral gap of the transition matrix **M**(*τ*) (i.e. the difference between the two largest eigenvalues, *λ*_1_ − *λ*_2_) which can be arbitrarily close to zero. This relation with the spectral gap is the key to understand why sudden transitions punctuate amply different stable states of the population while they do not entail a significant change in fitness. The spectral gap will be small in networks that show large heterogeneity, both in the degree distribution or in the weight associated to links and nodes. In the model presented here, this can be achieved only by (i) increasing the value of the lethality coefficient *f_l_*, which erases nodes and therefore includes heterogeneity in the degree, (ii) increasing the ruggedness of the fitness landscape *K*, which introduces heterogeneity in the weight of the nodes, or (iii) increasing the length of the sequences *N*, as it is known that large networks show sharper transitions^28^,^30^. In summary, the mutation-selection equilibrium appears as a complex interplay between the mutation rate, the local fitness of genomes and their correlations, and the global connectivity patterns. Local information is insufficient to predict the collective state of the population, which is summarised in the eigenvector 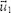. Also, the growth rate of the population at equilibrium (the effective population fitness given by *λ*_1_), has a value that does not coincide in general with a specific local or global maximum. That is to say, the sequence of highest fitness is not representative in general of the behaviour of the population.

**Figure 2:**
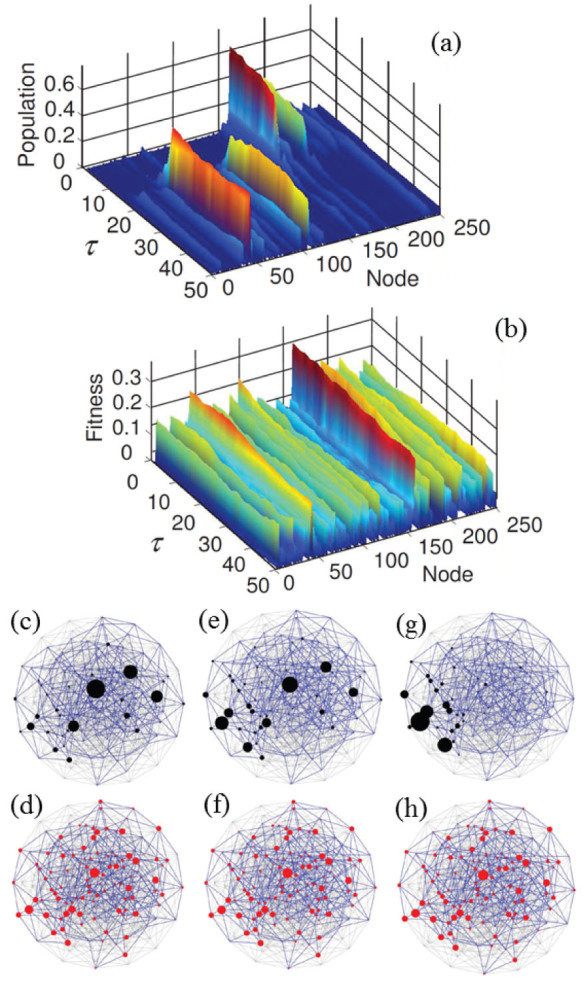
Description of a sudden shift in genomic space of a population responding to a stochastically varying environment. (a) Fraction of population (*u*_1_)*_i_*(*τ*) and (b) fitness of each genome *f_i_*(*τ*) for 50 successive environments. Genomes (or nodes in the space of genomes) are arbitrarily ordered along the *node*–axis; successive environments are listed in the *τ*–axis. (c, d) Before the genomic shift occurs (*τ* = 18), the most populated genome coincides with that of maximal fitness. (e, f) At the transition (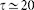), the population covers a broader region of the space of genomes. After the transition (*τ* = 22), the population is completely displaced to a new region (g), while changes in the fitness landscape are minor (h). The underlying space of genomes appears in pale grey; dark blue links connect genomes with positive fitness. Size of circles is proportional to sequence population (c, e, g) or to fitness (d, f, h). Parameters as in Fig. 1, and thus the size of the space of genomes is *A^N^* = 2^8^ = 256 nodes. See the Supplementary Video S1.

Finally, we analyse how the genomic population evolves over the space of genomes during a genomic shift. In order to study the phenomenon from this different perspective, Fig. 2 describes in detail a numerical example for a population of sequences evolving in a stochastically varying environment (same parameters as in Fig. 1). The fraction of population of each node -or sequence- *i* (given by the *i*-element of the eigenvector 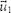 of the transition matrix **M**(*τ*), Fig. 2(a)) and its associated fitness (*f_i_*, Fig. 2(b)) are plotted for 50 subsequent environments. A sudden shift is observed in 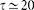, while no drastic change in fitness values is detected there. In Fig. 2 (c,e,g) and (d,f,h) the population and the fitness of each sequence are plotted over the space of genomes, in three critical situations: just before (*τ* = 18), during (*τ* = 20) and after (*τ* = 22) the tipping point. See the Supplementary Video S1 for an animated plot of this phenomenon.

### Driven fitness landscape

The second form in which the fitness landscape is varied in this work attends to the fact that changes in environmental conditions are often driven, showing a persistent increase or decrease in value superimposed to stochastic variations^2^,^8^. In natural systems, gradually changing environments have been shown to induce sudden shifts in population dynamics^31^,^32^. This situation can be implemented by generating two different fitness landscapes and interpolating linearly between the corresponding states of fitness for each genome. An additional parameter 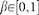 weights the relative contribution of each landscape.

The methodology is the following. As described in Methods, we generate a starting and an ending fitness landscape, 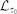 and 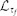, respectively, with the same parameters *N*, *K*, and *A*. For better visualisation of the process, and without loss of generality, we relabel the genomes such that those with maximum fitness in either landscape are at the largest Hamming distance. That is to say, genome (0, 0, 0, …) is assigned maximum fitness in the starting landscape by fixing 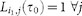, and sequence (1, 1, 1, …) has maximum fitness in the ending landscape by fixing 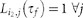, where *i*_1_ and *i*_2_ are the rows corresponding to *K* + 1 zeros and *K* + 1 ones respectively.

The elements of matrix **L***_τ_* corresponding to an intermediate landscape 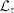 between *τ*_0_ and *τ_f_* become
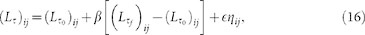


where parameter
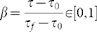
quantifies the linear transformation of the starting fitness landscape 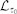 (corresponding to *β* = 0) into the ending landscape 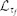 (*β* = 1), *η_ij_* is a random number uniformly distributed in [−1, 1], and 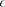 is the noise intensity. While noise is unnecessary to observe a genomic shift in this situation, it superimposes small fluctuations to the monotonic evolution of the fitness landscape, and allows for the estimation of several important quantities such as early warning signals based on such fluctuations. Once matrix **L***_τ_* is calculated, the fitness of every genome is obtained following the steps shown in Methods.

**Figure 3:**
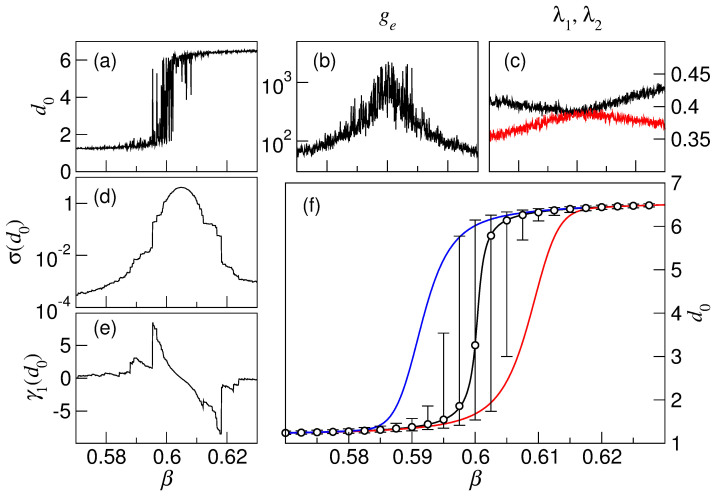
Early-warning signals and hysteresis in genomic shifts in a driven and stochastic fitness landscape. (a) Flickering appears around the transition, as measured by the genetic distance *d*_0_ of the population at equilibrium to genome 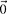. (b) Time to equilibrium *g_e_* dramatically increases close to the transition. (c) Eigenvalues *λ*_1_ (black) and *λ*_2_ (red) of the transition matrix. The closer they are, the larger *g_e_*. The spectral gap is typically large in regular networks^38^, while it can be arbitrarily close to zero in sufficiently large, heterogeneous networks. (d) Standard deviation *σ* and (e) skewness *γ*_1_ of *d*_0_ at equilibrium (averaged over 10^2^ consecutive landscapes). (f) The red and blue curves show the hysteretic behaviour of the genetic distance *d*_0_ of an out-of-equilibrium population subject to regular perturbations each *G* = 1 generations, for increasing and decreasing values of *β*. The black curve plots the genetic distances *d*_0_ at equilibrium for fixed *β* and 10^3^ realisations of the stochastic perturbations. Open circles represent the median of the distribution of *d*_0_ values; bars indicate the confidence interval 5–95%. Parameter *β* is sampled at intervals of Δ*β* = 10^−4^; other parameters are *N* = 8, *K* = 4, *f_l_* = 0.53, 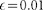 and *A* = 2.

Figure 3 shows a numerical genomic shift in a driven and stochastic fitness landscape that evolves as explained above. The abrupt transition occurs at a particular value of *β* around which stochasticity causes strong flickering, as can be seen in the evolution of the genetic distance *d*_0_ (Eq. (7), Fig. 3(a)). We have chosen the genetic distance to characterise this phenomenon because of its easy experimental measurement through deep-sequencing techniques (see below), but other quantities such as the average fitness of the population 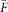 (Eq. (8)) would yield equivalent results. The time to reach mutation-seelection equilibrium *g_e_* grows significantly in the proximity of the transition (Fig. 3(b)). This quantity is larger the smaller the spectral gap *λ*_1_ − *λ*_2_, as can be inferred from Eq. (4). Due to stochasticity, *λ*_1_ and *λ*_2_ fluctuate around average values that become closer as the transition is approached (Fig. 3(c)), but note that the Perron-Frobenius theorem precludes *λ*_1_ = *λ*_2_ in networks of finite size^30^. For high and low values of *β*, far from the transition, the population is located in fairly stable and dissimilar states. Close to the transition, however, the stability of the two states is comparable, and they compete to attract the population. This is reflected in the standard deviation of the position of the population calculated as *σ*(*d*_0_) (Fig. 3(d)), which systematically increases as the tipping point is approached. An additional early-warning signal is skewness *γ*_1_(*d*_0_), the third standardised moment, a measure of the asymmetry in the fluctuations between the two states (Fig. 3(e)).

The dramatic increase in the time to equilibrium *g_e_* implies that, close to the transition, populations will not be able to attain high-fitness regions in the presence of too rapid environmental changes. A situation of fast change can be simulated by perturbing the landscape regularly, that is, increasing *β* every *G* generations. The concomitant delay in adaptation will show up when *G* is of the order of the time to equilibrium *g_e_* or smaller. The inability of the population to adapt before the new perturbation arrives (i.e. before the mutation-selection equilibrium is reached) causes hysteresis in its genomic composition, both for increasing and decreasing values of *β* (red and blue curves respectively in Fig. 3(f), where the genetic distance *d*_0_ of the population was plotted for *G* = 1).

In real cases, data to evaluate standard deviation and skewness of the genetic distance or any other experimental measure over several subsequent mutation-selection equilibria (varying *β*), may not be available. Instead, independent populations in similar environments might be at hand. In our model this would correspond to performing different realisations of the noise for each value of parameter *β*. Open circles in Fig. 3(f) represent the median of the distribution of *d*_0_ values obtained at equilibrium after 10^3^ realisations of the stochastic signal, and bars indicate the confidence interval, which reveals a strong asymmetry in the distribution. The dispersion of the state of the mutation-selection equilibria so obtained and their asymmetry also offer a clear signal that the transition is approaching.

Finally, note that the NK model yields a rugged landscape, devoid of strictly neutral regions, for any *K* ≥ 1^16^. In our scenario, it is analogous to an ensemble of quasi-neutral networks where neighbouring nodes are populated thanks to high mutation rates, complex local topology, and fitness correlations. However, analogous transitions are observed in holey-like fitness landscapes^26^,^27^,^33^, where large neutral regions are dominant. The early warning signals here used are easily applicable to that situation.

### Robustness of the model

Most assumptions of our model can be modified to simulate more realistic scenarios without causing qualitative changes in the phenomenology described. A full study of the generalisation of the results here presented is developed in section S2 of the Supplementary Information. We show in Supplementary Figure S1 that increasing the length of the genome, the degree of ruggedness of the landscape (or epistasis), the fraction of lethal mutations and the intensity of environmental changes, as well as decreasing the mutation rate, will increase the frequency and sharpness of transitions. Finally, we have not found major differences when the size of the alphabet *A* is varied: Some examples with *A* = 4 are studied, showing that also for alphabets of 4 letters (corresponding to RNA or DNA) increasing the length of the sequence, the ruggedness of the landscape and the fraction of lethal mutations enhance the intensity of transitions.

## Discussion

Once a transition occurs, it is very difficult for the system to return to its previous state^8^. Therefore, any measure yielding reliable information on the existence of a near tipping point and the concomitant warning signals is extremely valuable. Here we have proved that abrupt transitions at the genomic level preceded by early-warning signals are a generic phenomenon in constant and slowly driven landscapes affected by even slight stochasticity. We have used a very general model for the evolution of populations on fitness landscapes, and shown that most assumptions can be modified to implement a variety of scenarios without causing qualitative changes in the phenomenology described.

The only essential feature to observe sudden shifts in the genomic composition of a population is the existence of different regions in the landscape separated by fitness barriers, for which a non-negligible fraction of lethal mutations is a sufficient condition. Under environmental changes, the degree of adaptation attained by different genotypic clusters varies and sudden transitions set in. There are at least four features characteristic of some natural systems that actually enhance the abruptness of the transition. First, a higher degree of roughness of the landscape, which accentuates the effect of separating barriers. Second, a lower mutation rate in the population. Finite populations evolving at low mutation rates are less disperse in the space of genomes, even concentrating in a single genome if formed by identical individuals. These populations can explore the vicinity of the fitness landscape only close to the tipping point, and thus are expected to experience stronger hysteresis and more violent genomic shifts. Third, recombination might induce multiple equilibria and thus further separation into distinct genotypic clusters^34^. Fourth, a non-linear relationship between environment and fitness, as indirectly suggested by ecological extinctions caused by smooth environmental changes^35^. In such a case, also the genomic transition would be more severe, and eventually act as the ultimate cause of species extinction.

At larger scales, the controversial possibility of state shifts in the biosphere^8^,^9^ should be preceded by signals unfolding at a hierarchy of levels. At the lowest level, changes in the genomic composition of a population might predate population extinction, which might in turn affect the structure of ecosystems and lead to extinction cascades^36^. These effects could be subsequently transmitted bottom-up. The circle closes when realising that top-down effects might also occur under sudden climatic changes, which might preclude the adaptation of populations, as described. The extent to which the phenomenology presented here has implications at the ecological level and above deserves further exploration.

It is now possible to track the evolution of fast-mutating organisms, such as RNA viruses, through measures as simple as their consensus sequence, which corresponds to the most abundant nucleotide at each position. In controlled environments, rapid fixation of mutations (i.e. changes of nucleotides) in the consensus sequence might indicate that a genomic shift is taking place. Advances in deep sequencing open an interesting avenue to systematically probe the complex molecular structure of populations^37^, since this technique goes beyond consensus sequences to characterize single sequences actually present in the population. Quantitative measures of relative abundances of the different genotypes in a population yield a first approximation to the state eigenvector 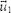, and might allow a characterisation of fitness landscapes and genotype network topology at an unprecedented level of detail. Joining measures of the growth rate of populations at equilibrium (corresponding to *λ*_1_) and estimations of the resilience of populations, now at hand^6^ (retrieving *λ*_2_), to predict the maximum approach of both quantities, should permit a first estimation of the time left before a genomic transition occurs. This could be of the maximum importance, as while early warnings can be used as an indicator of proximity of the tipping point, predicting the actual moment of the transition remains a challenge^2^.

At odds with other systems whose structure can be modified in order to avoid or regulate the appearance of critical transitions, genomic shifts may be unavoidable: the architecture of genotype networks is inherent to the chemical nature of life and its molecular organisation. A much deeper understanding of fitness landscapes and mutational mechanisms is needed before we can embark in the eventual control of genomic shifts. The artificial enhancement of barriers to adaptation or of the pace of environmental changes, even at the local scale, would be of the highest relevance to achieve that goal.

## Supplementary Material

Supplementary Information Supplementary Information

Supplementary Information Supplementary Video S1
