# Supplementary material for: Tipping points and early warning signals in the genomic composition of populations induced by environmental changes
Source: Sci Rep. 2015 May 12;5:9664. doi: 10.1038/srep09664 (PMC4428070; doi:10.1038/srep09664)
Supplement: Supplementary Information [file srep09664-s1.pdf]

Supplementary Information:

Tipping points and early warning signals in the genomic composition of populations induced by environmental changes, by J. Aguirre and S. Manrubia

Contents

S1 Calculation of the fitness value of a genome according to the NK model **2**

S2 Robustness of the model **3**

S2.1 Length of the genome . . . 3
S2.2 Ruggedness of the landscape (degree of epistasis) . . . 3
S2.3 Fraction of lethal mutations . . . 3
S2.4 Intensity of environmental noise . . . 4
S2.5 Mutation rate . . . 5
S2.6 Size of the alphabet . . . 5

S3 Analytical calculation of changes in population state and population fitness **6**

S3.1 Distribution of fitness changes for individual nodes . . . 6
S3.2 Distribution of changes in the population abundance of individual nodes . . . 6

S4 Supplementary Video S1. Animated description of a genomic shift in a population in a stochastically varying environment **9**

S1 Calculation of the fitness value of a genome according to the NK model

In this section, and for the sake of clarity, we present a detailed example of how to calculate the fitness of the genomes in a given fitness landscape $\mathcal{L}$, according to the NK model. We have focused on the fitness values f_s associated to the sequences s of length $N = 4$ loci, an alphabet of $A = 2$ letters (0 and 1) and landscape ruggedness $K = 1$. First, we construct the matrix $\mathbf{L}$ that represents fitness landscape $\mathcal{L}$. Its size is $A^{K+1} \times N = 4 \times 4$, and the elements L_{ij} are random numbers uniformly chosen between 0 and 1 (see Table S1 for a realization of such matrix).

	Locus 1	Locus 2	Locus 3	Locus 4
00	0.37	0.98	0.45	**0.62**
01	**0.85**	0.34	0.88	0.73
10	0.03	0.49	**0.25**	0.62
11	0.11	**0.59**	0.34	0.88

Table S1. Matrix $\mathbf{L}$ associated to fitness landscape $\mathcal{L}$ in the case of genomes of length $N = 2$, alphabet of $A = 2$ letters, and landscape ruggedness $K = 1$. Every locus j interacts with locus $j + 1$. Numbers in bold correspond to the contributions of the different loci to the total fitness of the genome $s = 0110$.

There are $A^{K+1} = 4$ values in each column, representing the A^{K+1} possible combinations of $K+1 = 2$ elements taking $A = 2$ different states (i.e. 00, 01, 10 and 11). For example, elements in column $j = 1$ (i.e. L_{11}, L_{21}, L_{31} and L_{41}) represent all possible contributions of the first locus of each genome to its total fitness. Index l_j stands for the correct one for locus j in a particular sequence.

In order to improve the comprehension of this methodology, let us calculate the fitness associated to sequence $s = 0110$. The actual contribution of locus j, $L_{l_j,j}$, depends on its state and on those of the $K = 1$ other loci that interact with it. In this case, we suppose that every locus j interacts with locus $j+1$ (and locus $j = N = 4$ interacts with locus 1), although any other criterion of interaction would be equally valid. For $s = 0110$ we obtain $l_1 = 2$, $l_2 = 4$, $l_3 = 3$ and $l_4 = 1$ (bold elements of matrix $\mathbf{L}$ in Table S1). Finally, the fitness of sequence $s = 0110$ is the average value of the fitness contributions of each of its loci,

$$f_{0110} = \frac{\sum_{j=1}^{N} L_{l_j,j}}{N} = \frac{0.85 + 0.59 + 0.25 + 0.62}{4} = 0.58\,. \tag{S1}$$

The calculation of the fitness associated to the remaining $A^N - 1 = 15$ sequences of length $N = 2$, alphabet of $A = 2$ letters, and ruggedness $K = 1$, in the fitness landscape $\mathcal{L}$ defined by Table S1, would be done in a similar fashion.

S2 Robustness of the model

In this section we examine several modifications of the main model with the aim of proving the robustness of the phenomenology described. We present results for the distribution of changes in the state of the population at equilibrium under a stochastically varying environment (see Methods). As mentioned, the presence of fat tails in these histograms is indicative of the non-linear response to changes in the landscape and the generality of genomic shifts.

S2.1 Length of the genome

Computational limitations prevent the systematic use of genome lengths much larger than those used here. Figure S1(a) compares two lengths, $N = 8$ and 12. There is a quantitative difference between the two response curves shown, the one with larger responses corresponding to the larger length. The amount of changes of small size in the population is depleted as N grows, while the tail of the distribution gains weight, meaning that more events of large size are found. Though relatively small in this example, this is a robust trend with sequence length. Increasing the length has indeed significant effects on the structure of fitness landscapes, causing an increase in the number of local fitness maxima among other structural changes [1]. In practice, increasing the length of the sequence works towards isolating different regions of the landscape, thus enhancing the magnitude of sudden transitions.

S2.2 Ruggedness of the landscape (degree of epistasis)

Figure S1(b) illustrates how different values of K (which controls epistasis and thus the ruggedness of the landscape) affect the response of the population. The larger K, the more likely are sudden changes. Only in the case of very low or no epistasis do we obtain a narrow response curve (exponential decay, dashed lines). A landscape without epistasis has a low degree of ruggedness and is therefore similar to a Fujiyama-like landscape, where dynamics of populations are smooth by construction. In our case, even small values of K cause the development of a fat tail (power-law decay, dotted lines) in the response curve.

S2.3 Fraction of lethal mutations

In the examples that illustrate this work, we have used several values of the lethality coefficient f_l. The reason is that in different systems, and also in populations with varying degrees of adaptation, the relative amount of beneficial, deleterious, and lethal mutations may vary amply [2]. Figure S1(c) illustrates the effect of parameter f_l in the dynamics of the model. There is a significant dependence of the strength of the response to environment on f_l. Actually, this turns out to be the parameter to which our model is most sensitive, yielding apparently narrow response curves (exponential decay, dashed lines) for small f_l and fat tails (power-law decay, dotted lines) for larger values.

As it is shown in Eq. (S12), and proved in the analytic calculations shown in section S3, it is the difference between the two largest eigenvalues, $\lambda_1 - \lambda_2$, that controls the intensity of change in the state of the population. It turns out that for too low values of f_l the number of sequences with fitness zero is very low and that difference typically does not come too close to zero when N is small, so changes are less pronounced [3]. However, our results when other variables are changed indicate that in realistic scenarios, where the degree of epistasis should be high, the sequences much longer than those that can be computationally tackled, or environmental perturbations much more frequent, low values of f_l and even $f_l = 0$ should also yield sudden changes in population states.

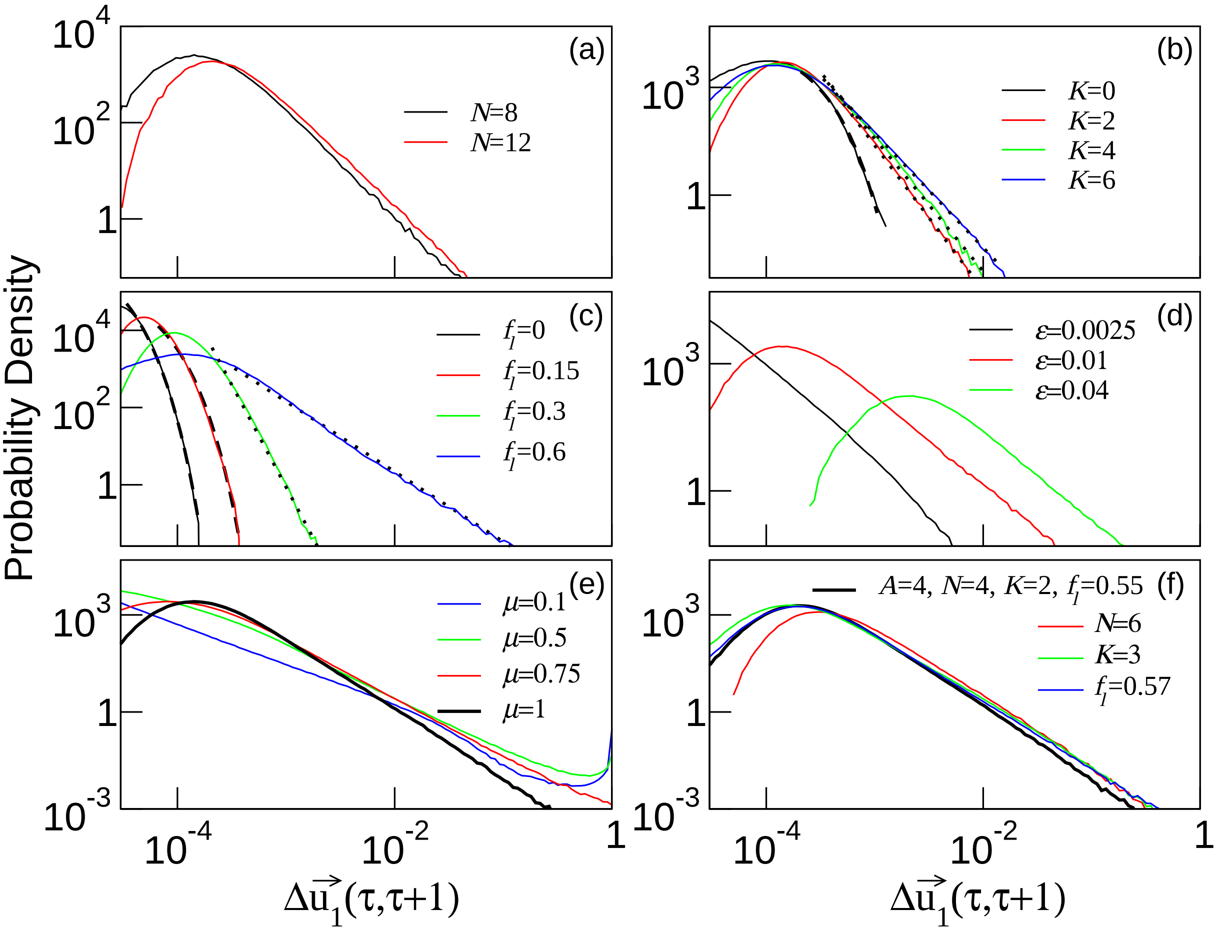

Figure S1. Distribution of changes in the mutation-selection equilibria of two populations at subsequent, stochastically varying environments. The curves plot the dependence of the difference between the population of subsequent equilibria $\Delta\vec{u}_1(\tau, \tau+1)$ with: (a) the length of the genome N, as shown in the legend; other parameters are $K = 4$, $A = 2$, $f_l = 0.5$ and $\epsilon = 0.01$; (b) the ruggedness (epistasis) K; other parameters are $N = 8$, $A = 2$, $f_l = 0.4$ and $\epsilon = 0.01$; (c) the lethality coefficient f_l; $N = 8$, other parameters as in a; (d) the environmental noise intensity ϵ; $N = 8$, other parameters as in a; (e) the mutation rate μ; $N = 8$, $K = 4$, $A = 2$, $f_l = 0.5$ ($\mu = 1$ is used throughout the paper); (f) the size of the alphabet; $A = 4$ and $\epsilon = 0.01$; the parameters of the bold black curve are shown in the legend; the rest maintain the same parameters, but increasing N (red curve), K (green), and f_l (blue). In (b) and (c), dashed lines represent exponential fits, and dotted lines power-law fits.

S2.4 Intensity of environmental noise

In most of our examples, except for the case of hysteresis, we have allowed the system to reach mutation-selection equilibrium before the next perturbation arrives. Also, we have selected noise of small amplitude. That is to say, our examples are very conservative regarding the pace of perturbations that populations might encounter in natural environments. Actually, it is even argued that fast-mutating populations subject to rapidly changing environments (such as RNA viruses, pathogens infecting different hosts, or free-living bacteria) are never able to attain true mutation-selection equilibria. Increases in the noise

intensity are one form in which such perturbations can be modelled. As shown in Fig. S1(d) and analytically described in Eq. (S2), the intensity of response of the population increases very significantly with our parameter ϵ. Finally, smaller values of ϵ than those used in these examples also yield the same phenomenology.

S2.5 Mutation rate

The transition matrix used in our dynamics, $\mathbf{M} = \mathbf{FG}$, is the particular case $\mu = 1$ of the more general situation $\mathbf{M} = r(1-\mu)\mathbf{FI} + \frac{r\mu}{S}\mathbf{FG}$ (Eq. (5) of the main text, see Methods for more information). In general, the mutation rate μ also plays a quantitative role in the dynamics, in addition to the fitness ($\mathbf{F}$) and topology ($\mathbf{G}$) of the landscape. In particular, μ may significantly affect the spread of the population over the genome space. In the limit of sufficiently small μ, all genomes might concentrate in a single node (homogeneous population) and this might severely interfere with the evolution of the population on the network [4]. In our case, however, this just enhances the intensity of the transitions, as shown in Fig. S1(e). As lower values of the mutation rate are used, the response curve displays an accumulation of abrupt transitions between non-overlapping population states (at $\Delta\vec{u}_1(\tau, \tau+1) \sim 1$). Independently of the value of μ used, the typical response curve presents a fat tail over several decades.

S2.6 Size of the alphabet

Finally, in Figs. 1-3 of the main text we have considered binary sequences (a two-letter alphabet), while in the case of nucleotides the size of the alphabet would be 4, rising to 20 in the case of proteins, and occasionally larger and certainly variable in the case of genes and their alleles. We can see in Fig. S1(f) that there are no major differences when the size of the alphabet is varied. To do so, we have plotted in bold black the curve corresponding to a typical case with $A = 4$, while the rest maintain the same parameters, but increasing N (red curve), K (green), and f_l (blue). The shift shown in all three cases towards larger values of the difference between subsequent equilibria $\Delta\vec{u}_1(\tau, \tau+1)$ certifies that increasing the length of the sequence, the ruggedness of the landscape and the fraction of lethal mutations enhance the intensity of transitions: in summary, that the general results obtained for 2-letter alphabets are also applicable to larger alphabets.

S3 Analytical calculation of changes in population state and population fitness

It is possible to calculate how the variations in the fitness of a node and in its population are distributed as the environment changes. The results, that we develop in this section, provide a proof of the qualitative difference between both distributions: the stochastic variable characterizing changes of fitness in a single node has a Gaussian, narrow distribution, while the response of the population of that node to changes in fitness can be arbitrarily large. Hence, the distribution corresponding to the latter variable can develop a long, fat tail. For clarity, and without any loss of generality, in these calculations we will assume $f_l = 0$ (no lethal mutations), and a stochastically varying fitness landscape (see Methods).

S3.1 Distribution of fitness changes for individual nodes

Consider the stochastic variable X_i, which describes the changes in the fitness value of node i in contiguous environments, $X_i = f_i(\tau+1) - f_i(\tau)$.

Let us recall how matrix $\mathbf{L}$, which eventually defines f_i, changes from τ to $\tau+1$ under a stochastically fluctuating fitness landscape,

$$L_{jk}(\tau+1) = L_{jk}(\tau) + \epsilon\eta_{jk}(\tau)\,,$$

where $L_{jk}(\tau)$ are random numbers uniformly distributed in $[0,1]$, $\eta_{jk}(\tau)$ are random numbers uniformly distributed in $[-1,1]$ and ϵ is the strength of the noise. Hence, following Eq. (1) of the main manuscript,

$$f_i(\tau+1) = \frac{\sum_{k=1}^{N}[L_{l_k^i,k}(\tau) + \epsilon\eta_{l_k^i,k}(\tau)]}{N}$$

and

$$X_i(\tau) = f_i(\tau+1) - f_i(\tau) = \frac{\epsilon}{N}\sum_{k=1}^{N}\eta_{l_k^i,k}(\tau)\,.$$

Since $\epsilon\eta_{l_j,k}$ is a random variable uniformly distributed in $[-\epsilon,\epsilon]$, its mean is zero and its variance equals $\epsilon^2/3$. In the limit of large N we can apply the Central Limit Theorem, obtaining that the probability distribution of the stochastic variable $X_i(\tau) = f_i(\tau+1) - f_i(\tau)$ follows a Gaussian distribution with mean and variance

$$\begin{aligned} E[X] &= E[f_i(\tau+1) - f_i(\tau)] = 0\,, \\ Var(X) &= Var(f_i(\tau+1) - f_i(\tau)) = \frac{\epsilon^2}{3N}\,. \end{aligned} \tag{S2}$$

S3.2 Distribution of changes in the population abundance of individual nodes

In this section we calculate the statistical properties of the changes in the fraction of sequences populating each node under an environmental change from τ to $\tau+1$, similarly to the previous section. To this end, we will consider the stochastic variable $Y_i = (u_1)_i(\tau+1) - (u_1)_i(\tau)$.

Using the results in section S3.1, we can estimate the change in the elements of the transition matrix $\mathbf{M}$ as

$$M_{ij}(\tau+1) = f_i(\tau+1)G_{ij} = M_{ij}(\tau) + X_i(\tau)G_{ij}\,,$$

or in matrix form

$$\mathbf{M}(\tau+1) = \mathbf{F}(\tau+1)\mathbf{G} = \mathbf{M}(\tau) + \mathbf{X}(\tau)\mathbf{G} = \mathbf{M}(\tau) + \frac{\epsilon}{\sqrt{3N}}\mathbf{P}\,,$$

where $\mathbf{X}(\tau) = \mathbf{F}(\tau+1) - \mathbf{F}(\tau)$ and $\mathbf{P}$ is a matrix whose entries are distributed as a Gaussian, $\mathcal{N}(0,1)$. Therefore, the calculation of the changes in the population of a node with time reduces to the study of the perturbation of the transition matrix $\mathbf{M}$ by another matrix [5] whose elements are in this case taken from a Gaussian distribution of mean zero and standard deviation $\epsilon/\sqrt{3N}$.

We need, therefore, the expression of the eigenvector associated to the largest eigenvalue of a perturbed non-symmetric, non-negative matrix. For the sake of completeness, we show its analytical derivation.

Proposition 1. *Let* $\mathbf{M}$ *be a non-symmetrical and non-negative matrix of size* $m \times m$*, eigenvalues* λ_i*, right eigenvectors* $\vec{u}_i$ *and left eigenvectors* $\vec{u}_i^L$*. Let us perturb* $\mathbf{M}$ *in a way such that* $\mathbf{M}' = \mathbf{M} + \epsilon\mathbf{Q}$*, where* $\epsilon \ll 1$ *and* $\mathbf{Q}$ *is a perturbation matrix whose elements* Q_{ij} *are arbitrary. The first eigenvalue* λ_1' *of the perturbed matrix* $\mathbf{M}'$ *and its associated eigenvector* $\vec{u}_1'$ *can be expanded as*

$$\begin{aligned}
\lambda_1' &= \lambda_1 + \epsilon \vec{u}_1^L \mathbf{Q} \vec{u}_1 + \epsilon^2 \sum_{k=2}^{m} \frac{(\vec{u}_1^L \mathbf{Q} \vec{u}_k)(\vec{u}_k^L \mathbf{Q} \vec{u}_1)}{\lambda_1 - \lambda_k} + o(\epsilon^3)\,, \\
\vec{u}_1' &= \vec{u}_1 + \epsilon \sum_{k=2}^{m} \frac{\vec{u}_k^L \mathbf{Q} \vec{u}_1}{\lambda_1 - \lambda_k} \vec{u}_k + o(\epsilon^2)\,.
\end{aligned}$$

Proof. The main target of the analytical calculation shown here is to describe the new matrix $\mathbf{M}'$ as a perturbation of matrix $\mathbf{M}$, and consequently obtain the maximum eigenvalue λ_1' of $\mathbf{M}'$ as a perturbation of λ_1, and its associated eigenvector $\vec{u}_1'$ as a perturbation of $\vec{u}_1$. This methodology is inspired on the perturbation theory of matrices shown in [6], and has been applied in the context of Complex Network theory [5].

By definition

$$\mathbf{M}' \vec{u}_1' = \lambda_1' \vec{u}_1'\,, \tag{S3}$$

where

$$\begin{aligned}
\mathbf{M}' &= \mathbf{M} + \epsilon \mathbf{Q}, & \text{(S4)} \\
\vec{u}_1' &= \vec{u}_1 + \epsilon \vec{v}_1 + \epsilon^2 \vec{v}_2 + o(\epsilon^3), & \text{(S5)} \\
\lambda_1' &= \lambda_1 + \epsilon t_1 + \epsilon^2 t_2 + 0(\epsilon^3). & \text{(S6)}
\end{aligned}$$

We first substitute Eqs. (S4), (S5) and (S6) in Eq. (S3) and equate terms with the same order of ϵ. Taking into account that $\parallel \vec{u}_1' \parallel = 1 \Rightarrow \vec{u}_1 \cdot \vec{v}_1 = 0$, we obtain to first order in ϵ

$$\begin{aligned}
\vec{u}_1^L \cdot (\mathbf{M}\vec{v}_1 + \mathbf{Q}\vec{u}_1) &= \vec{u}_1^L \cdot (\lambda_1 \vec{v}_1 + t_1 \vec{u}_1) \\
\Rightarrow t_1 &= \vec{u}_1^L \mathbf{Q} \vec{u}_1 \\
\Rightarrow (\mathbf{M} - \lambda_1)\vec{v}_1 &= (\vec{u}_1^L \mathbf{Q} \vec{u}_1 - \mathbf{Q})\vec{u}_1\,, & \text{(S7)}
\end{aligned}$$

(where the superindex L indicates the left eigenvector), and to second order, ϵ^2

$$\begin{aligned}
\vec{u}_1^L \cdot (\mathbf{M}\vec{v}_2 + \mathbf{Q}\vec{v}_1) &= \vec{u}_1^L \cdot (\lambda_1 \vec{v}_2 + t_1 \vec{v}_1 + t_2 \vec{u}_1) \\
\Rightarrow t_2 &= \vec{u}_1^L \mathbf{Q} \vec{v}_1.
\end{aligned}$$

Vector $\vec{v}_1$ can be written as

$$\vec{v}_1 = \sum_{k=1}^{m} c_k \vec{u}_k\,. \tag{S8}$$

Including Eq. (S8) in (S7), and multiplying both sides by $\vec{u}_k^L$ from the left, we obtain $c_k = \frac{\vec{u}_k^L \mathbf{Q} \vec{u}_1}{\lambda_1 - \lambda_k}$ for $k > 1$. Since $\vec{u}_1 \cdot \vec{v}_1 = 0$, $c_1 = 0$. All this yields

$$\vec{v}_1 = \sum_{k=2}^{m} \frac{\vec{u}_k^L \mathbf{Q} \vec{u}_1}{\lambda_1 - \lambda_k} \vec{u}_k \,, \tag{S9}$$

and including Eq. (S9) in Eq. (S5), and Eq. (S8) in Eq. (S6), we finally obtain

$$\begin{aligned} \lambda_1' &= \lambda_1 + \epsilon \vec{u}_1^L \mathbf{Q} \vec{u}_1 + \epsilon^2 \sum_{k=2}^{m} \frac{(\vec{u}_1^L \mathbf{Q} \vec{u}_k)(\vec{u}_k^L \mathbf{Q} \vec{u}_1)}{\lambda_1 - \lambda_k} + o(\epsilon^3)\,, \\ \vec{u}_1' &= \vec{u}_1 + \epsilon \sum_{k=2}^{m} \frac{\vec{u}_k^L \mathbf{Q} \vec{u}_1}{\lambda_1 - \lambda_k} \vec{u}_k + o(\epsilon^2)\,. \end{aligned}$$

Note that $\vec{u}_1^L \mathbf{Q} \vec{u}_k = \sum_{i,j}^{m} (u_1)_i^L Q_{ij} (u_k)_j$ and $\vec{u}_k^L \mathbf{Q} \vec{u}_1 = \sum_{i,j}^{m} (u_k)_i^L Q_{ij} (u_1)_j$.

□

Let us return to our particular case. Making use of the Eq. (S3) just obtained, and taking into account that $m = A^N$ and $\mathbf{Q} = \frac{1}{\sqrt{3N}} \mathbf{P}$, for each node i we obtain

$$(u_1)_i(\tau+1) = (u_1)_i(\tau) + \frac{\epsilon}{\sqrt{3N}} \sum_{l=2}^{A^N} \sum_{jk \in \{conn\}} \frac{\alpha_{ijkl}}{\lambda_1 - \lambda_l} P_{jk} + o(\epsilon^2)\,,$$

where $\alpha_{ijkl} = (u_l^L)_j (u_1)_k (u_l)_i$, P_{jk} are random numbers normally distributed following $\mathcal{N}(0,1)$ and $\{conn\}$ is the set of pairs jk corresponding to connected pairs of nodes of the space of genomes.

If we sum a number of Gaussian variables with different variances, the result is also normally distributed and its variance is equal to the sum of such variances. Therefore, it follows

$$E[Y_i] = E[(u_1)_i(\tau+1) - (u_1)_i(\tau)] \sim \frac{\epsilon}{\sqrt{3N}} \sum_{l=2}^{A^N} \sum_{jk \in \{conn\}} \frac{\alpha_{ijkl}}{\lambda_1 - \lambda_l} E[P_{jk}] = 0\,, \tag{S10}$$

$$\begin{aligned} Var(Y_i) = Var((u_1)_i(\tau+1) - (u_1)_i(\tau)) &\sim \frac{\epsilon^2}{3N} \sum_{l=2}^{A^N} \sum_{jk \in \{conn\}} \left(\frac{\alpha_{ijkl}}{\lambda_1 - \lambda_l} \right)^2 Var(P_{jk}) = \\ &\frac{\epsilon^2}{3N} \sum_{l=2}^{A^N} \sum_{jk \in \{conn\}} \left(\frac{\alpha_{ijkl}}{\lambda_1 - \lambda_l} \right)^2 . \end{aligned} \tag{S11}$$

Note that, as mentioned in Methods and proved in [7], the eigenvalues of matrix $\mathbf{M}$ are real. Therefore, the terms $l = 2$ in the summations in Eqs. (S10) and (S11) are the most relevant ones because $\lambda_1 - \lambda_l > \lambda_1 - \lambda_2$ for $l > 2$. Therefore, we finally obtain

$$Var((u_1)_i(\tau+1) - (u_1)_i(\tau)) \propto \frac{\epsilon^2}{N(\lambda_1 - \lambda_2)^2}\,, \tag{S12}$$

from where we see that the perturbations suffered by the population of each node after one environmental change can be arbitrarily high when λ_2 becomes close to λ_1.

Finally, note that, as $Var(f_i(\tau+1) - f_i(\tau)) = \epsilon^2/(3N)$,

$$\frac{Var((u_1)_i(\tau+1) - (u_1)_i(\tau))}{Var(f_i(\tau+1) - f_i(\tau))} = \frac{Var(Y_i)}{Var(X_i)} \propto (\lambda_1 - \lambda_2)^{-2}\,.$$

S4 Supplementary Video S1. Animated description of a genomic shift in a population in a stochastically varying environment

The evolution of the genomic composition shown in Figs. 2(a), (c), (e), (g) and the evolution of the fitness landscape shown in Figs. 2(b), (d), (f) and (h) of the main text are plotted in Supplementary Video S1. The underlying regular space of genomes is plotted in pale grey, while dark blue links connect sequences with positive fitness. The size of each node i is proportional to population of sequence i, $(u_1)_i$, (plotted in white) and to its fitness f_i (plotted in red). As in Fig. 2 of the main text, 50 values of τ were plotted. Parameters are: length of the sequence $N = 8$ (which leads to a sequence space of 256 nodes), landscape ruggedness $K = 4$, alphabet $A = 2$, lethality coefficient $f_l = 0.55$, and noise intensity $\epsilon = 0.01$.

References

1. Kauffman, S. A. & Levin, S. Towards a general theory of adaptive walks on rugged landscapes. *Journal of Theoretical Biology* **128**, 11–45 (1987).

2. Stich, M., Lazaro, E. & Manrubia, S. C. Phenotypic effect of mutations in evolving populations of RNA molecules. *BMC Evolutionary Biology* **10**, 46 (2010).

3. Donetti, L., Neri, F. & Muñoz, M. A. Optimal network topologies: expanders, cages, ramanujan graphs, entangled networks and all that. *Journal of Statistical Mechanics* **2006**, P08007 (2006).

4. Huynen, M. A., Stadler, P. F. & Fontana, W. Smoothness within ruggedness: the role of neutrality in adaptation. *Proceedings of the National Academy of Sciences USA* **93**, 397–401 (1996).

5. Aguirre, J., Papo, D. & Buldú, J. M. Successful strategies for competing networks. *Nature Physics* **9**, 230–234 (2013).

6. Marcus, R. Brief comments on perturbation theory of a nonsymmetric matrix: The GF matrix. *Journal of Physical Chemistry A* **105**, 2612–2616 (2001).

7. Aguirre, J., Buldú, J. M. & Manrubia, S. C. Evolutionary dynamics on networks of selectively neutral genotypes: Effects of topology and sequence stability. *Physical Review E* **80**, 066112 (2009).
